# Hypothermia-induced accelerated idioventricular rhythm after cardiac surgery; a case report

**DOI:** 10.1186/s12872-023-03178-y

**Published:** 2023-03-20

**Authors:** Saeid Hosseini, Soheila Salari, Sepideh Banar, Yousef Rezaei, Atieh Tajik, Ali Zahedmehr, Zahra Emkanjoo

**Affiliations:** 1 Heart Valve Diseases Research Center, Rajaie Cardiovascular Medical and Research Center, Iran University of Medical Sciences, Tehran, 1995614331 Iran; 2 Cardiovascular Intervention Research Center, Rajaie Cardiovascular Medical and Research Center, Iran University of Medical Sciences, Tehran, 1995614331 Iran; 3 Cardiac Electrophysiology Research Center, Rajaie Cardiovascular Medical and Research Center, Iran University of Medical Sciences, Tehran, 1995614331 Iran

**Keywords:** Accelerated Idioventricular Rhythm, Coronary Artery Bypass Grafts, Cardiac Surgery, Hypothermia

## Abstract

**Background:**

Accelerated idioventricular rhythm (AIVR) is a slow ventricular arrhythmia, commonly due to myocardial ischemia in coronary artery disease. It is a transitory rhythm that rarely causes hemodynamic instability or necessitates any specific therapy. Besides, the common predisposing factors for ventricular arrhythmias after open-heart surgery are hemodynamic instability, electrolyte imbalances, hypoxia, hypovolemia, myocardial ischemia and infarction, acute graft closure, reperfusion injury, and administration of inotropes and antiarrhythmic drugs. Here we report a case of AIVR after cardiac surgery, mostly due to hypothermia that to our knowledge, it is the first report.

**Case presentation:**

We describe a 76-year-old man presenting with typical chest pain. Following routine investigations, the patient underwent coronary artery bypass grafting. Postoperatively, he was transferred to the intensive care unit with good hemodynamic status. However, about 3 h later, he developed rhythm disturbances, leading to hemodynamic instability without response to volume replacement or inotropic support. His rhythm was AIVR, although, at first glance, it resembled the left bundle branch block. Given his unstable hemodynamic status, he was emergently transferred to the operating room. Cardiopulmonary bypass (CPB) was resumed for hemodynamic support. After the patient was rewarmed to about 35 ºC, AIVR returned to normal. He was weaned from CPB successfully and with an uneventful hospital course.

**Conclusions:**

Hypothermia is a potential cause of rhythm disturbance. Preventing the causes of arrhythmias, including hypothermia, is the best strategy.

**Supplementary Information:**

The online version contains supplementary material available at 10.1186/s12872-023-03178-y.

## Background

Cardiac rhythm disturbances after coronary artery bypass graft (CABG) surgery have been reported in approximately one-third of patients [1, 2]. Many studies have evaluated contributing factors among patients undergoing cardiac surgeries with the implementation of cardiopulmonary bypass (CPB). The development of rhythm disturbances is associated with several factors, including a previous history of inferior myocardial infarction, right coronary artery stenosis, left main coronary artery stenosis, the maximal temperature of the left circumflex artery region, the aortic cross-clamp time, hypertension, and some medications [2–6]. Moreover, damage to the atrioventricular node during valve surgeries is a major cause of postoperative conduction disturbances [7].

The nature of cardiac surgeries, which are usually performed with patient cooling and cold cardioplegia, may result in injury to the conduction system [8]. Hypothermia may cause arrhythmias after cardiac surgeries [9]. Herein, we describe a patient who underwent CABG and was then weaned from CPB with good hemodynamic status and in the sinus rhythm but, in the early hours of arrival at the intensive care unit (ICU), developed accelerated idioventricular rhythm (AIVR) due to hypothermia, causing a state of shock. The patient recovered after rewarming with the aid of CPB.

## Case presentation

**Fig. 1:**
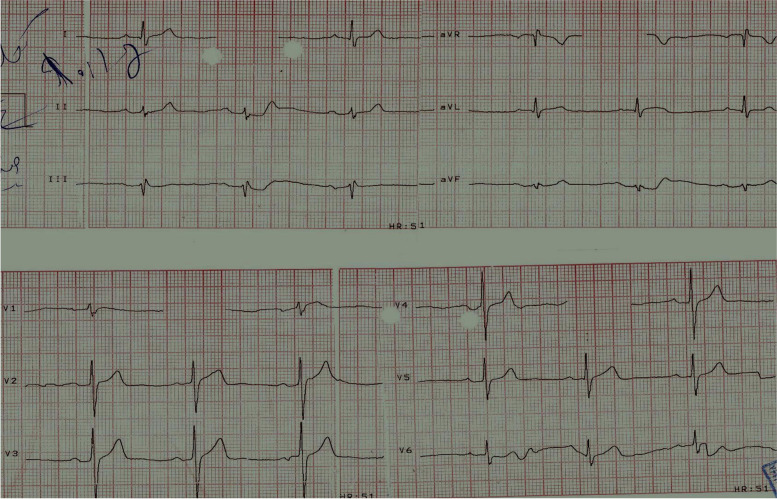
The electrocardiogram shows a normal sinus rhythm at baseline before first surgery

A 76-year-old man came to the hospital with typical chest pain, suggestive of ischemic heart disease. Coronary angiography showed a triple-vessel coronary artery disease. Transthoracic echocardiography revealed a left ventricular ejection fraction (EF) of 50%. Electrocardiography (ECG) showed a normal sinus rhythm (Fig. 1). The patient took loading dose of clopidogrel (600 mg) during angiography 10 days earlier and then continued 75 mg daily till 3 days before surgery. He underwent a 4-grafts CABG surgery( left internal mammary artery on left anterior descending and 3 saphenous vein grafts on Diagonal, obtuse marginal and right coronary artery). Myocardial protection was done with repeated doses of tepid hyperkalemic blood cardioplegia ( Buckberg cardioplegia) in total 1700 cc in 4 interrupted doses. Duration of the aortic cross-clamp time and CPB time was 74 and 120 min, respectively. The lowest temperature during CPB was 30 ºC. After the successful weaning of the patient from CPB in normothermia(37 c), hemostasis was done.

His hemostasis and then sternal closure took longer than usual due to the residual antiplatelet effect of clopidogrel**.**. Subsequently, he was transferred to the ICU with good hemodynamic status and in the sinus rhythm.

**Fig. 2:**
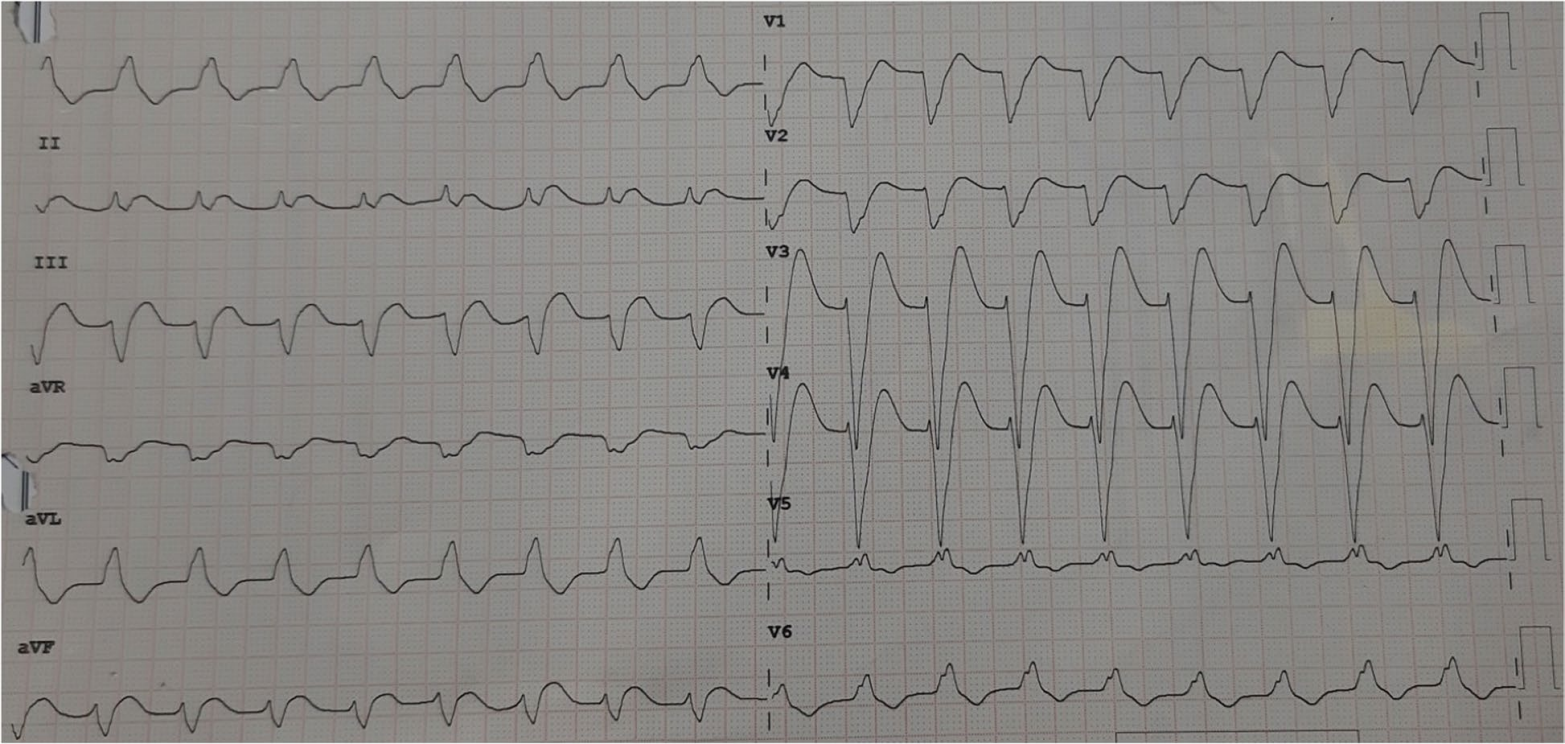
The electrocardiogram shows an accelerated idioventricular rhythm after CABG in ICU

After ICU entrance, despite good hemodynamic and diuresis during first 3 h, his ECG suddenly changed an accelerated AIVR (Fig. 2), and the patient experienced hypotension unresponsive to volume and inotropic medication. Due to hemodynamic instability, he was emergently transferred to the operating room. CPB was started again, and an intra-aortic balloon pump was inserted. The bypass graft vessels (saphenous vein graft and left internal mammary artery) seemed open (pinkish color of the saphenous grafts and good pulsatility of the internal mammary artery). Transesophageal echocardiography revealed a left ventricular EF of 50%, and the other findings were the same as those before the operation. The patient’s temperature was 31 ºC. When his temperature rose to about 35 °C, AIVR reversed to a normal baseline ECG, as first seen on the anesthesia monitor and immediately confirmed by taking a 12-lead ECG.

**Fig. 3:**
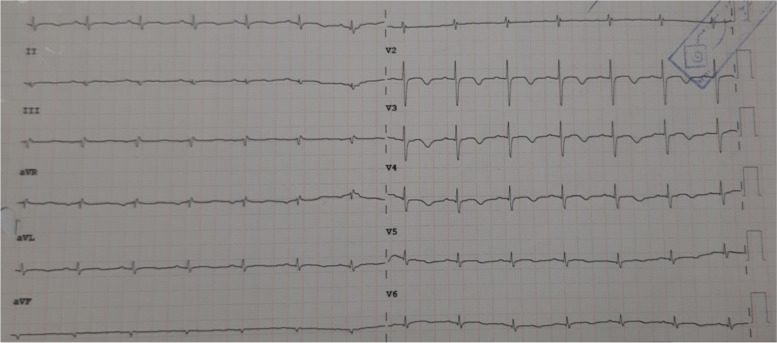
The electrocardiogram shows the disappearance of the arrhythmia

With complete rewarming to normothermia(37 ºC), the patient was weaned from CPB uneventfully with a mild dose of an inotropic medication.After hemostasis and chest-wall closure, he was referred to the ICU, where he remained hemodynamically stable without any arrhythmia (Fig. 3). After 5 days, he was discharged from the hospital with stable hemodynamic status and good clinical features. At 3 months’ follow-up, he had a stable condition without any conduction disturbances (Table 1).

**Table 1:** Electrolytes and Acid Base data and hemodynamic data

	**Operation room data before leaving of the main surgery**	**ICU data on arrival after the main surgery**	**ICU data before come back to operation room again**	**Operation room data on starting the 2**^**nd**^** operation**	**Operation room data before transfer to ICU again**
T	**37**	**Not mentioned**	**Not mentioned**	**32.2**	**37.1**
PH	**7.49**	**7.25**	**7.30**	**7.39**	**7.36**
PCO_2_	**34**	**40**	**32**	**30**	**38**
HCO_2_	**25**	**19**	**16**	**18**	**21**
BE	**3**	**-8**	**-8**	**-6**	**-3**
NA	**141**	**145**	**142**	**135**	**146**
K	**3.9**	**4.5**	**3.9**	**4.4**	**4.5**
HB	**11.7**	**9.2**	**8.4**	**8.4**	**9.2**
HCT	**38**	**29**	**27**	**27**	**28**
PLT	**280,000**	**260,000**	**280,000**	**260,000**	**250,000**
MEAN BP	**97.5 mmgh**	**77.5 mmgh**	**77.5 mmgh**	**60.5 mmgh**	**97.5 mmgh**

## Discussion

The ECG manifestations of hypothermia include the J (Osborn) wave; prolonged PR, QRS, and QT intervals; and atrial and ventricular arrhythmias. Hypothermia decreases the spontaneous depolarization of cardiac pacemaker cells, prolongs the action potential duration of both depolarization and repolarization, and slows myocardial impulse conduction. However, the incidence of conduction disturbances is not common with hypothermia [4].

On the other hand, CABG surgery is associated with arrhythmias, including atrial fibrillation, ventricular arrhythmias, and conduction disturbances [5, 6]. The predisposing factors of myocardial ischemia, such as severe coronary artery disease, preoperative myocardial infarction, and transient ischemia, are also associated with postoperative conduction disturbances [10, 11]. Although on-pump CABG usually provides excellent protection of the myocardium with the help of the cardioplegic solution, poor myocardial protection could be a major contributor to postoperative arrhythmias [12].

The main features of the cardioplegic solution that may lead to the development of cardiac rhythm disturbances consist of its type, temperature (cold vs warm) [8], administration route (antegrade, retrograde, or combined) [13], and potassium concentrations [14].

Reduced body temperatures can result in diminished metabolic rates and cardiovascular functions and, subsequently, decreased pacemaker and conduction velocity, rendering patients prone to arrhythmias. Hypothermia-induced arrhythmias consist of bradycardia, heart block, and prolonged QT intervals [15]. Postoperative conduction disturbances are mainly self-limiting or may only require temporary pacing [6, 16].

In this case, the patient developed rhythm disturbances and, thus, hypotension around 3 h after ICU entrance following CABG surgery. At first glance, this arrhythmia resembled the left bundle branch block; nonetheless, meticulous evaluations of the patient’s clinical and ECG points led us to a diagnosis of AIVR. The mechanisms involved in AIVR development mainly include abnormal calcium-dependent automatism foci, which are seen in acute myocardial infarction or reperfusion periods, digitalis-induced events, some types of cardiomyopathies, and the presence or absence of structural heart diseases in children [17, 18].

The pathophysiology of ventricular arrhythmia development in hypothermic hearts remains unknown. The most common reported ventricular arrhythmia in the setting of hypothermia is ventricular fibrillation (VF). Experimental studies from canine wedge preparations have shown that conduction block and reentrant VF during rewarming are associated with transmural and epicardial repolarization dispersion [19, 20]. Combined with slowed conduction velocity at 30 °C in rabbit hearts, these circumstances may favor unidirectional block and the induction [21].

Generally, surgical team love lower operation room temperature due to heating of surgical gown wearing and heating of the surgical lights. Although there are some heating maneuver to overcome hypothermia of the patients as warming blanket but in longer than usual operation time, hypothermia would be possible.

Gurabi Z et al [22] demonstrated the effect of hypothermia in accentuating repolarization abnormalities within the left ventricular epicardium in the setting of the emergency department. This effect of hypothermia is accentuated, leading to the development of phase II reentry and VT/VF. The mechanism of AIVR in hypothermia might be explained by the circus movement theory. The main factor responsible for the initiation of a circus movement type of tachycardia is an increase in the conduction time/refractory period ratio.

Accordingly, we posit that our patient’s AIVR must have been caused by hypothermia because it disappeared after his rewarming. Indeed, there were no other causes for the occurrence of this arrhythmia like electrolyte abnormalities or ischemia.

## Conclusions

Arrhythmias after cardiac operations could occur, although they are mostly self-limiting. The phenomenon of hypothermia and its impact on prognosis should be considered in patients undergoing open-heart surgeries.

## Supplementary Information

**Additional file 1.** Video ligand.

## Data Availability

The datasets used and/or analyzed during the current study are available from the corresponding author on reasonable request.

## Abbreviations

CABG: Coronary artery bypass graft
AIVR: Accelerated idioventricular rhythm
CPB: Cardiopulmonary bypass
ECG: Electrocardiography
EF: Ejection fraction
ICU: Intensive care unit
VF: Ventricular fibrillation
VT: Ventricular tachycardia
