# Supplementary figures and images for: Hypothermia-induced accelerated idioventricular rhythm after cardiac surgery; a case report
Source: BMC Cardiovasc Disord. 2023 Mar 20;23:142. doi: 10.1186/s12872-023-03178-y (PMC10026505; doi:10.1186/s12872-023-03178-y)

**Video ligand:**

**Video-1: Coronary angiography**

**
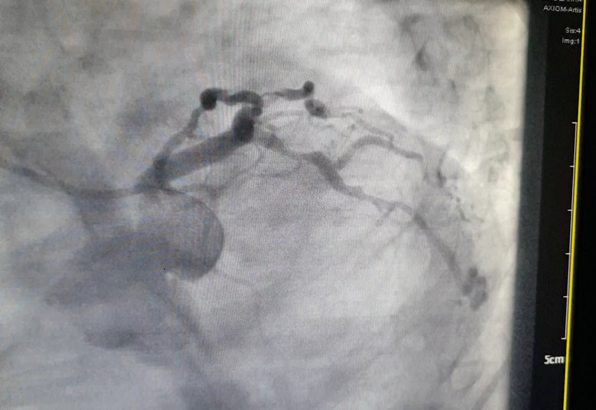

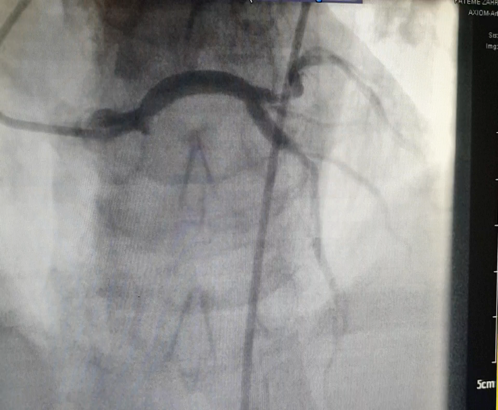

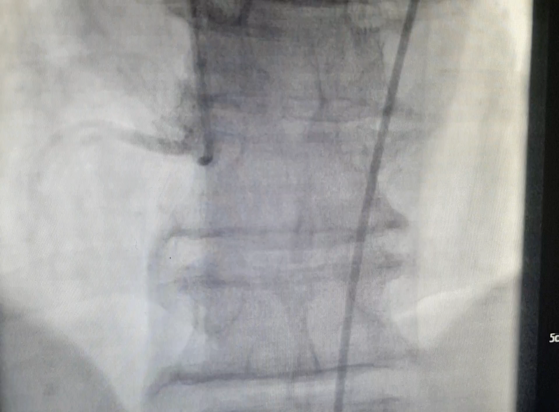
**

**Video 2: Echocardiography**

**
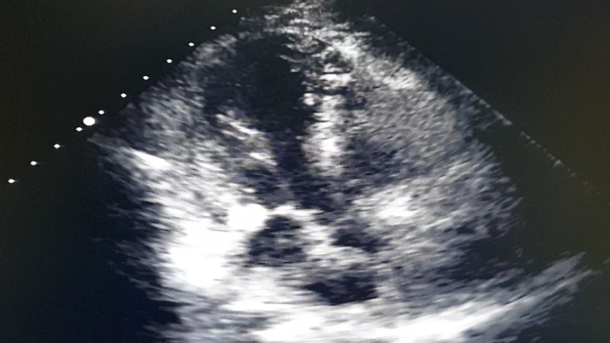

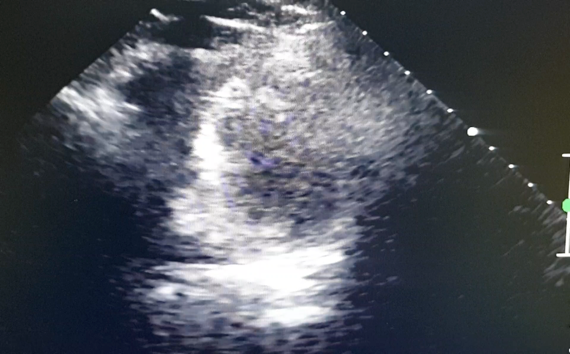

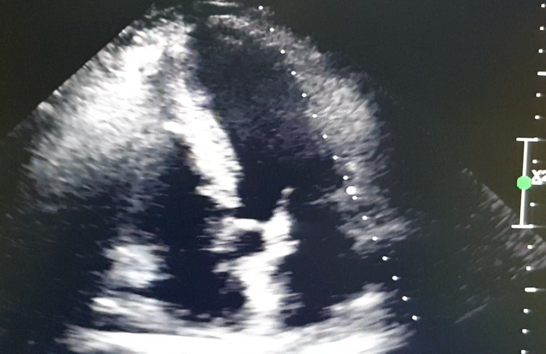
**

**
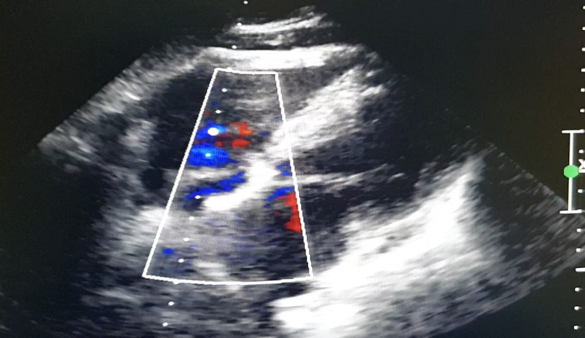

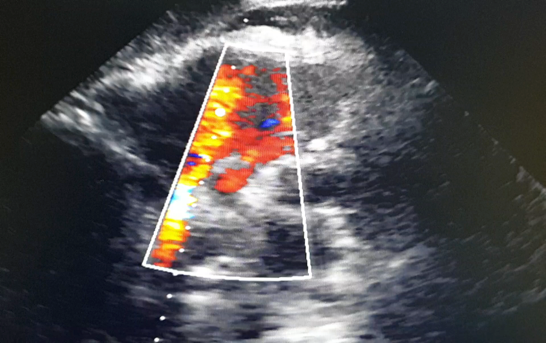
**

Supplement: Supplementary file 1 — Additional file 1. Video ligand. [file 12872_2023_3178_MOESM1_ESM.docx]
